# Overproduced *Brucella abortus *PdhS-mCherry forms soluble aggregates in *Escherichia coli*, partially associating with mobile foci of IbpA-YFP

**DOI:** 10.1186/1471-2180-10-248

**Published:** 2010-09-28

**Authors:** Charles Van der Henst, Caroline Charlier, Michaël Deghelt, Johan Wouters, Jean-Yves Matroule, Jean-Jacques Letesson, Xavier De Bolle

**Affiliations:** 1 Molecular Biology Research Unit (URBM), University of Namur (FUNDP), 61 rue de Bruxelles, 5000 Namur, Belgium; 2 Theoretical and Structural Physical Chemistry Unit (UCPTS), University of Namur (FUNDP), 61 rue de Bruxelles, 5000 Namur, Belgium

## Abstract

**Background:**

When heterologous recombinant proteins are produced in *Escherichia coli*, they often precipitate to form insoluble aggregates of unfolded polypeptides called inclusion bodies. These structures are associated with chaperones like IbpA. However, there are reported cases of "non-classical" inclusion bodies in which proteins are soluble, folded and active.

**Results:**

We report that the *Brucella abortus *PdhS histidine kinase fused to the mCherry fluorescent protein forms intermediate aggregates resembling "non-classical" inclusion bodies when overproduced in *E. coli*, before forming "classical" inclusion bodies. The intermediate aggregates of PdhS-mCherry are characterized by the solubility of PdhS-mCherry, its ability to specifically recruit known partners fused to YFP, suggesting that PdhS is folded in these conditions, and the quick elimination (in less than 10 min) of these structures when bacterial cells are placed on fresh rich medium. Moreover, soluble PdhS-mCherry foci do not systematically colocalize with IpbA-YFP, a marker of inclusion bodies. Instead, time-lapse experiments show that IbpA-YFP exhibits rapid pole-to-pole shuttling, until it partially colocalizes with PdhS-mCherry aggregates.

**Conclusion:**

The data reported here suggest that, in *E. coli*, recombinant proteins like PdhS-mCherry may transit through a soluble and folded state, resembling previously reported "non-classical" inclusion bodies, before forming "classical" inclusion bodies. The dynamic localization of IbpA-YFP foci suggests that the IbpA chaperone could scan the *E. coli *cell to find its substrates.

## Background

*Escherichia coli *is widely used to produce recombinant proteins of interest. One of the major concerns in the overproduction process is the formation of insoluble structures called inclusions bodies (IB) [1,2]. IB formation results from the aggregation of misfolded polypeptides that have escaped quality control by chaperones and proteases to interact through their exposed hydrophobic regions before precipitating [3]. Aggregate formation and features are influenced by various growth conditions such as temperature and pH [4], culture phase [5] and glucose/oxygen availability [6].

*In vivo *protein aggregation is a dynamic reversible process [7]. Chaperones involved in aggregate dissociation, e.g. DnaK/DnaJ/ClpB and IbpA/IbpB, colocalize with IB in *E. coli *[8-11]. Recently, it has been reported that aggregate cellular localization is not random [9]. Small protein aggregates are delivered to a cell pole to form larger structures that are further dissolved by an energy dependent process [12]. All proteins in IB were initially considered as unfolded, but it has been shown that some polypeptides inside aggregates are present in an active form [2,13,14]. Several groups reported the formation of "non-classical" IB mainly characterized by the presence of folded and soluble recombinant proteins [15,16].

Here, we report a novel example of "non-classical" IB that contain folded and soluble recombinant proteins and only transiently interact with the IpbA chaperone. Indeed, overproduction of *Brucella abortus *PdhS cytoplasmic histidine kinase [17] in *E. coli *revealed that PdhS-mCherry fusions were first folded and soluble in aggregates formed during the stationary phase of culture before forming insoluble structures having all the characteristics of "classical" IB. These "classical" IB recruited IpbA-YFP, as previously reported for other IB in *E. coli *[11], unlike the intermediate "non classical" IB. We observed that IbpA-YFP was able to form foci with very dynamic properties inside *E. coli *and to reach and colocalize with soluble PdhS-mCherry aggregates.

## Results

### PdhS-mCherry forms growth phase-dependent aggregates in *E. coli*

**Figure 1:**
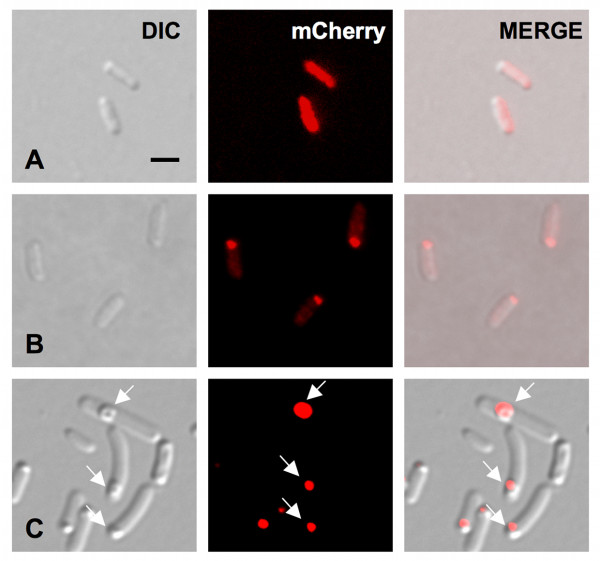
**Fluorescent distribution of PdhS-mCherry fusion in stationary growth phase *E. coli***. **A**, early stationary phase; **B**, middle stationary phase; **C**, late stationary phase. White arrows point to refractile bodies that are only present in the bacteria from the late stationary culture phase. Scale bar: 2 μm. DIC means differential interference contrast (Nomarski). All micrographic images were taken with the same magnification.

We used the pCVDH07 plasmid to overexpress the *pdhS *coding sequence (CDS) fused in frame with the CDS for the fluorescent reporter mCherry (see Materials and Methods). Interestingly, the localization of this fusion in *E. coli *revealed foci at quarter (2%), mid-cell (10%) and polar (88%) sites of *E. coli *S17-1 in the stationary phase (n = 200) (Fig. 1B). The PdhS-mCherry was a stable fusion in *E. coli*, since Western blot analysis using antibodies raised against mCherry revealed a major band with the expected molecular mass for the complete fusion (data not shown). Fusing the *pdhS *CDS to the *yfp *or *cfp *CDS on the same backbone plasmid or overexpressing the *pdhS-mCherry *fusion in DH10B, TOP10 and MG1655 *E. coli *strains also generated similar fluorescent foci (data not shown). When a *pdhS-mCherry *fusion was carried on a low-copy plasmid, there was no polar focus in *E. coli*, contrary to its expression in *B. abortus *where PdhS-mCherry monopolar foci were present (data not shown). Other *B. abortus *proteins (the DivK response regulator, FumA and FumC fumarases) fused to the mCherry N-terminus did not generate fluorescent foci but rather a diffuse signal (data not shown). Taken together, this data suggests that foci formation in *E. coli *is mainly due to PdhS itself and to the abundance of the whole PdhS-mCherry recombinant protein.

Given that bacteria growth conditions strongly influence aggregate formation, we checked whether the fluorescent foci were dependent on the growth phase, as previously reported for IB [5]. Using the *pdhS-mCherry *overexpressing strain, we observed bacteria grown until the early, mid and late stationary phase, corresponding to bacteria having just reached the maximal turbidity of the culture (t_0_), the bacteria 12 h later (t_12_), and the bacteria 36 h later (t_36_), respectively. At t_0 _of the stationary culture phase, very few bacteria (4%, n = 100) showed polar fluorescent foci as many were associated with a bright diffuse cytoplasmic fluorescent signal (Fig. 1A). Twelve hours later in the same medium (t_12_), polar fluorescent foci were observed (in 98% of the observed bacteria, n = 100), together with a decrease of the diffuse cytoplasmic fluorescent signal (Fig. 1B). No detectable refractile bodies were observed in these conditions. After 24 additional hours (t_36_), larger and brighter fluorescent polar foci were formed, colocalizing with dense refractile bodies typical of "classical" IB, and accompanied by a strong decrease of the diffuse fluorescent signal (Fig. 1C).

**Figure 2:**
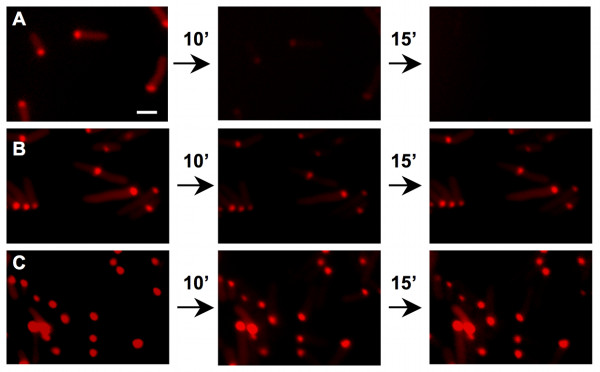
**Stability of PdhS-mCherry aggregates in *E. coli* grown until the stationary culture phase**. Fluorescent micrographic images taken using TxRed filter to visualize mCherry fluorescence. Pictures were taken using the same parameters, at intervals of 10 and 15 min, as indicated. **A**, middle stationary phase bacteria on agarose pad supplemented with LB medium; **B**, middle stationary phase bacteria on agarose pad with PBS; **C**, late stationary phase on LB medium. Scale bar: 2 μm. All micrographic images were taken with the same magnification.

When stationary phase bacteria (at t_12_) showing polar fluorescent PdhS-mCherry aggregates were placed on an agarose pad made with rich medium (LB), fluorescent structures quickly disappeared (in less than 10 minutes) (Fig. 2A). Interestingly, when bacteria of the same culture were placed on an agarose pad containing phosphate buffered saline (PBS), fluorescent foci were still detectable for 3 hours and even increased in size and intensity with the appearance of dense refractile bodies (Fig. 2B). Fluorescence decrease in rich medium did not result from photobleaching, since fluorescence was still detectable after repeat exposure of bacteria on agarose pads without additional rich medium. The "classical" IB present in late stationary phase bacteria (at t_36_) were still observable when these bacteria were placed on an agarose pad supplemented with LB rich medium (Fig. 2C) or PBS (data not shown). Together, these data suggest that fluorescent foci observed during the mid stationary phase are reversible and different from those observed during the late stationary phase of culture.

### Colocalization assays between PdhS-mCherry fluorescent aggregates and IbpA-YFP fusions

IbpA (for Inclusion body protein A) is a small heat shock chaperone discovered in *E. coli *[8]. The IbpA-YFP fusion was already successfully used to label inclusion bodies *in vivo*, in single cells of *E. coli *[11]. As PdhS-mCherry fluorescent polar foci generated during the mid and late stationary culture phases could differ from each other, we tested their possible colocalization with the IbpA-YFP fusion.

**Figure 3:**
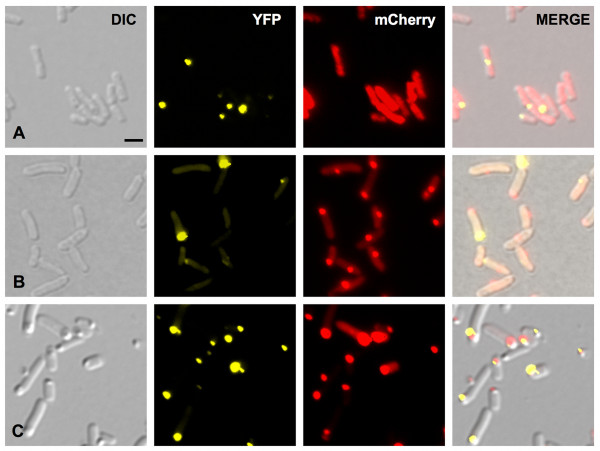
**IbpA-YFP and PdhS-mCherry localization pattern in stationary culture phase *E. coli***. **A**, early stationary phase; **B**, middle stationary phase; **C**, late stationary phase. Pictures were taken with Normarski (DIC), as well as YFP and mCherry typical fluorescence. The same parameters were applied for each culture condition. Scale bar: 2 μm. All micrographic images were taken with the same magnification.

**Figure 4:**
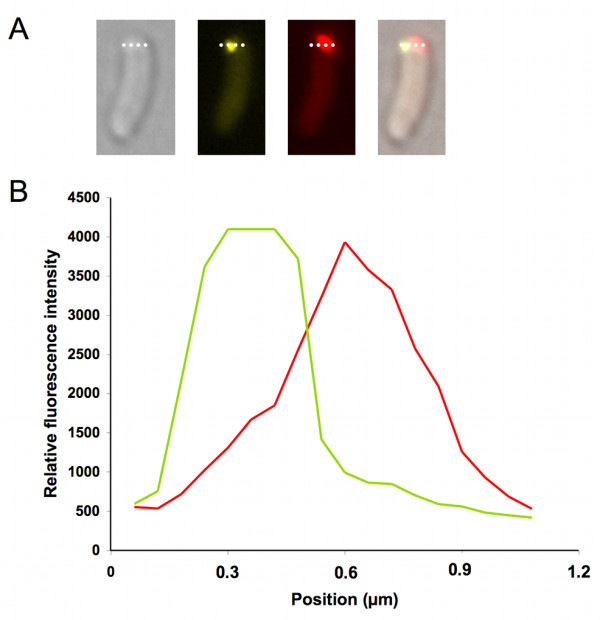
**IbpA-YFP and PdhS-mCherry colocalization pattern in stationary culture phase *E. coli***. **A**, Partial colocalization of IbpA-YFP and PdhS-mCherry. Relative fluorescent intensity was computed along the dotted white bar. **B**, Distribution of relative fluorescent signal as shown in A. In green, fluorescent distribution of IbpA-YFP signal. In red, PdhS-mCherry fluorescent signal.

We transformed the pCVDH07, to overexpress the *pdhS-mCherry *fusion, in a strain expressing a chromosomal *ibpA-yfp *fusion, previously used to monitor aggregates *in vivo *[11]. Using fluorescence microscopy, we observed the PdhS-mCherry aggregates and IbpA-YFP localization in early, mid and late stationary phase bacteria (Fig. 3). During the early stationary phase (t_0_), the bacteria displayed a diffuse cytoplasmic PdhS-mCherry signal while IbpA-YFP foci were mainly present at the cell poles (Fig. 3A). Surprisingly, in mid stationary phase bacteria (t_12_), colocalization of PdhS-mCherry with IbpA-YFP was quite rare (Fig. 3B). Indeed, only 15% of these bacteria (n = 250) displayed the two corresponding fluorescent foci at the same poles, 15% at the opposite pole, 15% at an intermediate position (often near midcell) and, in 60% of these bacteria, only one fluorescent focus corresponding to PdhS-mCherry was detectable. Moreover, in the bacteria with both fluorescent signals at the same pole, we systematically observed that PdhS-mCherry and IbpA-YFP did not exactly overlap (Fig. 4). At a later stage of the stationary culture phase (t_36_), polar colocalization of PdhS-mCherry and IbpA-YFP was frequent (78%) (Fig. 3C), but the signal of both fluorescent fusions was also slightly shifted. In these late stationary phase bacteria, both foci also colocalized with dense refractile bodies seen in differential interference contrast (DIC) (Fig. 3C). At t_36_, the polar IbpA-YFP foci were more frequent and were larger and brighter compared with non-polar IbpA-YFP foci. Western blot analyses showed that the IbpA-YFP fusion was not cleaved (data not shown).

**Figure 5:**
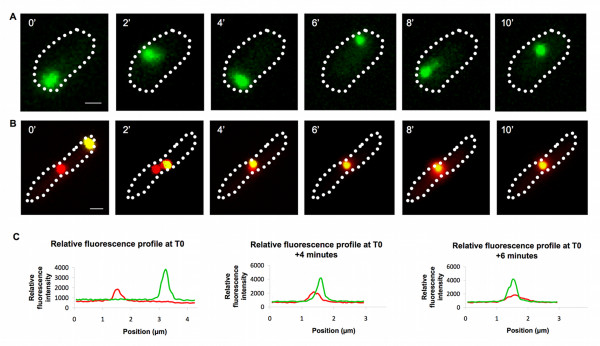
**Dynamic localization pattern of IbpA-YFP in stationary growth phase *E. coli***. Fluorescent micrographic images of middle stationary phase bacteria plated on rich medium taken every 2 minutes. A: IbpA-YFP; B: IbpA-YFP (yellow) and PdhS-mCherry (red). C: Fluorescence intensity of IbpA-YFP (green) and PdhS-mCherry (red) fusions at times T0, T0+4 minutes and T0+6 minutes. Scale bar: 1 μm

Time-lapse experiments were performed to monitor the kinetics of the cytoplasmic distribution of PdhS-mCherry and IbpA-YFP fusions. Mid stationary growth phase bacteria (t_12_) were plated on LB agarose pads and observed every two minutes at 37°C (see Materials and Methods). We observed a very dynamic localization pattern of IbpA-YFP foci in bacteria that did not contain a PdhS-mCherry aggregate (Fig. 5A). In contrast, when the PdhS-mCherry aggregate was present in t_12 _bacteria, IbpA-YFP foci moved from pole to pole until they colocalized with the immobile PdhS-mCherry foci (movie S1, Fig. 5B and 5C), which in turn progressively disappeared, as previously observed (Fig. 2). In the late stationary phase cultures, the large IbpA-YFP polar clusters colocalizing with PdhS-mCherry did not move (data not shown).

### PdhS-mCherry fusions in fluorescent foci of mid stationary phase cells display properties of folded proteins

Since the PdhS-mCherry foci observed during the mid stationary phase did not colocalize with IbpA-YFP, it was tempting to speculate that PdhS-mCherry fusions were correctly folded in these aggregates. In keeping with this idea, Western blot analysis using anti-mCherry antibodies showed that PdhS-mCherry was mainly found in the soluble fraction of bacteria grown until the late stationary phase (Additional file 2, Figure S1). When soluble extracts were examined by gel permeation combined with fluorescence and Western blot analysis, soluble PdhS-mCherry proteins were identified as a single peak, with a predicted molecular weight between 669 kDa and 20,000 kDa, the upper limit of the fractionation range (Additional file 2, Figure S2). This suggests that the fusion is able to form multimers with a defined number of monomers, further implying that PdhS-mCherry is folded.

**Figure 6:**
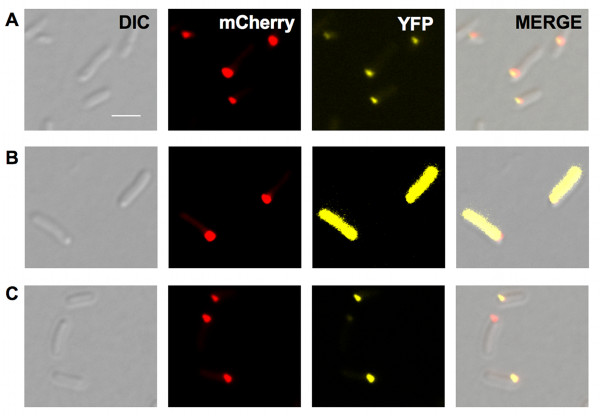
**PdhS-mCherry fusion is still able to recruit known partners**. PdhS-mCherry localization with (A) *B. abortus *FumC-YFP, (B) *Caulobacter crescentus *FumC-YFP, and (C) *B. abortus *DivK-YFP. Bacteria were cultivated until middle stationary culture phase. Scale bar: 2 μm. All micrographic images were taken with the same magnification.

Using yeast two-hybrid assays, it was recently shown that *B. abortus *PdhS was able to interact with FumC through its amino-terminal domain [18], and with DivK through its carboxy-terminal domain [17]. Interestingly, FumC from *Caulobacter crescentus *did not interact with *B. abortus *PdhS [18]. When *B. abortus *FumC-YFP and DivK-YFP fusions were produced with PdhS-mCherry, colocalization of YFP and mCherry fluorescence signals was observed in mid stationary phase *E. coli *cells (Fig. 6A, C). Interestingly, both fluorescence signals were overlapping, further suggesting that the shift in fluorescence signals observed between PdhS-mCherry and IbpA-YFP (Fig. 4) was not an artefact. As a control, we checked that *C. crescentus *FumC did not colocalize with PdhS-mCherry (Fig. 6B). The ability of PdhS-mCherry to recruit *B. abortus *DivK-YFP and FumC-YFP but not *C. crescentus *FumC-YFP suggests that the N-terminal and C-terminal domains of PdhS were at least partially folded.

## Discussion

We report that, when overproduced in *E. coli*, *B. abortus *PdhS fused to mCherry is able to form intermediate aggregates of soluble proteins resembling previously reported "non-classical" IB [3,15], before forming "classical" IB. These intermediate aggregates are very different from "classical" IB because they are soluble, are quickly removed when bacteria are placed in rich medium (Fig. 2A), do not systematically colocalize with IbpA-YFP (Fig. 3B) and are still able to recruit known PdhS partners (Fig. 6). The observation of "intermediate" aggregates of soluble proteins does not fit with a simple model of IB formation in which unfolded proteins precipitate to form IB immediately after translation. Our observations thus suggest that some proteins could form aggregates of folded and soluble polypeptides before their precipitation into "classical" IB. The initial solubility of heterologous PdhS-mCherry could be due to a slow expression (as suggested by the slow accumulation of PdhS-mCherry, Additional file 2, Figure S1), since there is no predicted promoter identified upstream the CDS in the pCVDH07 plasmid, and the codon bias of *pdhS *CDS is probably not optimal for sustained translation. Strong accumulation could lead to the saturation of chaperones and proteolysis activities, explaining the slow transition between soluble and "classical" IB.

The data we report suggests that PdhS-mCherry is folded in aggregates resembling "non-classical" IB. The data supporting the folded state of PdhS in *E. coli *are that PdhS-mCherry (i) is soluble and forms multimers of homogeneous size, and (ii) is still able to interact with partners like the fumarase FumC and the response regulator DivK. The recent resolution of a complex between a histidine kinase and its cognate response regulator [19] strongly suggests that the dimerization and histidine-containing phosphotransfer (DHp) domain of the kinase needs to be folded to allow interaction with the response regulator. It is therefore predictable that at least the DHp domain of PdhS-mCherry is folded to allow interaction with DivK-YFP. Interestingly, we previously reported that *B. abortus *PdhS was able to colocalize with *B. abortus *fumarase FumC, but not with *C. crescentus *FumC [18], and here the recruitment of FumC proteins by PdhS-mCherry is consistent with this specificity (Fig. 6A and 6B). Moreover, it means that fusions to YFP are not all aspecifically associated to soluble aggregates of PdhS-mCherry resembling "non-classical" IB.

A striking observation is the mobility of IbpA-YFP foci inside cells during the stationary phase (at t_12_). This mobility is strongly decreased in late stationary cells (t_36_), where larger and brighter IbpA-YFP foci are observed at the bacterial poles. IbpA-YFP foci also move around in PdhS-mCherry aggregates producing cells at t_12_, until they meet PdhS-mCherry aggregates. The dynamic localization of IbpA-YFP suggests a model in which IbpA could scan the bacterial cell to bind to protein aggregates before taking part in a disaggregation process. This hypothesis is supported by the observation of a fading of PdhS-mCherry fluorescence when it colocalizes with IbpA-YFP, concomitantly with an increase of the diffuse mCherry fluorescent signal (Fig 5C, Additional File 1), suggesting that a fraction of PdhS-mCherry is removed from the "non-classical" IB. It would be interesting to test whether IbpA-YFP dynamic intracellular distribution is dependent on cytoskeletal elements. It would also be interesting to colocalize the IbpB co-chaperone with IbpA, and to investigate the role of the IbpA fibrils [20] in the intracellular motion of IbpA. Indeed, IbpA fibril formation is inhibited by aggregated substrates [20], and here we observed that IbpA-YFP is moving until it reaches IB. The absence of systematic colocalization of IbpA-YFP with PdhS-mCherry (Fig. 3B) suggests that IbpA does not tightly and systematically bind all types of protein aggregates in *E. coli*. Even when IbpA-YFP localizes to the same pole as PdhS-mCherry, the position of the two foci is clearly distinct (Fig. 4), compared to the overlap observed for PdhS-mCherry and DivK-YFP or *B. abortus *FumC-YFP (Fig. 6). This suggests that IbpA-YFP and PdhS-mCherry do not truly colocalize, like PdhS-mCherry with DivK-YFP or FumC-YFP, which have been reported to directly bind to PdhS [17,18].

## Conclusion

PdhS-mCherry is a new example of a protein able to form soluble "non-classical" inclusion bodies in *E. coli*. Here we report a detailed characterization of these particular IB using several approaches. These IB are able to recruit partners of PdhS, suggesting that PdhS remains folded in these IB, at least during a first step of IB maturation. The "non-classical" IB are probably highly sensitive to proteolysis, since they are quickly cleared from the cells when the environmental conditions change. Time lapse analysis of *E. coli *cells containing PdhS-mCherry "non-classical" IB indicates that IbpA-YFP foci move rapidly inside the bacteria until they reach fluorescent aggregates. The characterization of IbpA-YFP movement inside *E. coli *should be investigated further as it could indicate how the IbpA chaperone is able to scan the cytoplasm to recognize intracellular protein aggregates.

## Methods

### Strains, plasmids and media

*E. coli *strains MG1655 expressing the *ibpA *coding sequence (CDS) fused to the enhanced version of YFP CDS (13) and S17-1, TOP10 and DH10B were grown in liquid Luria-Bertani (LB) broth medium at 37°C. Antibiotics were used at the following concentrations when appropriate: kanamycin, 50 μg/ml and chloramphenicol, 20 μg/ml. The *pdhS *CDS was inserted in fusion with the *mCherry *CDS on a high-copy number plasmid, in the opposite orientation of the *lac *promoter, derived from the pBluescriptKS vector (Stratagene); this plasmid was named pCVDH07. The *E. coli *strains transformed with pCVDH07 were grown in liquid LB with kanamycin for times indicated in the text, without induction of gene expression for the PdhS-mCherry fusion. The growth was followed by measuring the optical density at 600 nm.

### Microscopy

For fluorescence imaging, *E. coli *S17-1 and MG1655 strains were placed on a microscope slide that was layered with 1% agarose containing either PBS or 1% agarose containing LB medium (40 g/l). Time-lapse microscopy was performed by placing strains on a microscope slide that was layered with a 1% agarose pad containing LB medium. Fluorescence corresponding to the mCherry reporter was observed at 583 nm using a TxRed filter. Fluorescence corresponding to the YFP signal was observed using an emission filter centered on 535 nanometers and an excitation from 490 to 510 nanometers. Samples were observed every 2 min using a Nikon i80 fluorescence microscope and the NIS software from Nikon with a Hamamatsu camera.

### Protein extracts and Western blotting

Cultures at the mid stationary phase (optical density at 600 nm of 1.5) were centrifuged and then washed twice in 20 mM Tris-HCl 100 mM NaCl buffer at pH 7.9, lysed by sonication carried out over periods of 30 s with 1 min intervals in cooled tube on ice using a Branson sonifier 150. The cells were disrupted as observed microscopically to obtain total bacterial lysates that were centrifuged for 15 minutes at 13,000 rpm at 4°C. After centrifugation, the supernatant was harvested and considered as the soluble fraction of the bacterial cell lysate. The pellet was resuspended in PBS to reach the same volume as the supernatant, and was considered as the insoluble fraction. The soluble and insoluble fractions were then analysed by Western blot using polyclonal anti-DsRed antibodies (Clontech Laboratories, Inc) recognizing the mCherry protein, as previously reported (16).

### Gel filtration

The soluble fraction of bacterial lysate (500 μl) was injected into a HiPrep 16/60 Sephacryl S-500 HR column (GE Healthcare). The calibration curve was obtained using thyroglobulin (669 kDa), apoferritin (443 kDa) and amylase (200 kDa). One milliliter fractions were collected and tested for the presence of the mCherry fluorochrome using a fluorimeter equipped with a TxRed filter. Positive fluorescent fractions were then tested by Western blot analysis using anti-DsRed antibodies.

## Authors' contributions

CVDH performed all experiments with the help of others, as indicated below, and drafted the manuscript. CC and JW performed to the gel permeation experiment. MD participated to the construction of the plasmid used for PdhS-mCherry production in *E. coli*. JYM contributed to the microscopy. JJL participated in the writing of the manuscript. XDB coordinated the study and finalized the manuscript. All authors read and approved the final manuscript.

## Supplementary Material

Additional file 1 **Movement of IbpA-YFP in *E. coli* cells producing PdhS-mCherry.** Time lapse movie of* E. coli *cells at stationary (t_12_) phase, producing PdhS-mCherry (red) and IbpA-YFP (yellow). The time interval between two pictures is 2 min. Click here for file

Additional file 2 **Time course of PdhS-mCherry production and gel permeation analysis of soluble extracts.** PdhS-mCherry recombinant protein is detected by Western blot in the soluble fraction of *E. coli* expressing *pdhS-mCherry* fusion, and in the insoluble fraction in cells at late stationary phase (Figure S1). Western blot and fluorescence were used to detect PdhS-mCherry in gel permeation fractions, and allow the identification of a single peak corresponding to this fusion (Figure S2). Click here for file
