# Supplementary material for: Overproduced Brucella abortus PdhS-mCherry forms soluble aggregates in Escherichia coli, partially associating with mobile foci of IbpA-YFP
Source: BMC Microbiol. 2010 Sep 28;10:248. doi: 10.1186/1471-2180-10-248 (PMC2957392; doi:10.1186/1471-2180-10-248)
Supplement: Additional file 2 — Time course of PdhS-mCherry production and gel permeation analysis of soluble extracts. PdhS-mCherry recombinant protein is detected by Western blot in the soluble fraction of E. coli expressing pdhS-mCherry fusion, and in the insoluble fraction in cells at late stationary phase (Figure S1). Western blot and fluorescence were used to detect PdhS-mCherry in gel permeation fractions, and allow the identification of a single peak corresponding to this fusion (Figure S2). [file 1471-2180-10-248-S2.PDF]

Supplementary figures

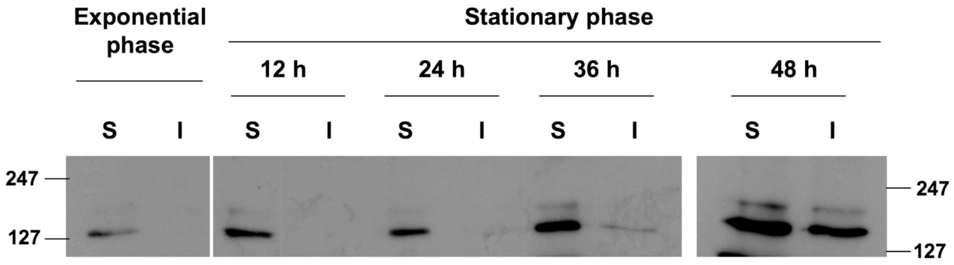

Figure S1. Western blot analysis of soluble and insoluble fractions of *E. coli* expressing PdhS-mCherry fusion. The soluble and insoluble fractions are indicated by "S" and "I" letters above the lanes, respectively. The time spent in stationary culture phase is also indicated. The expected molecular weight of the fusion is 146 kDa, and position of the molecular weight markers of 127 kDa and 247 kDa are indicated. Anti-DsRed antibodies were used as described in Methods to detect mCherry. The material loaded in each lane corresponds to 20 microL of culture at an optical density of 2.0 at 600 nm.

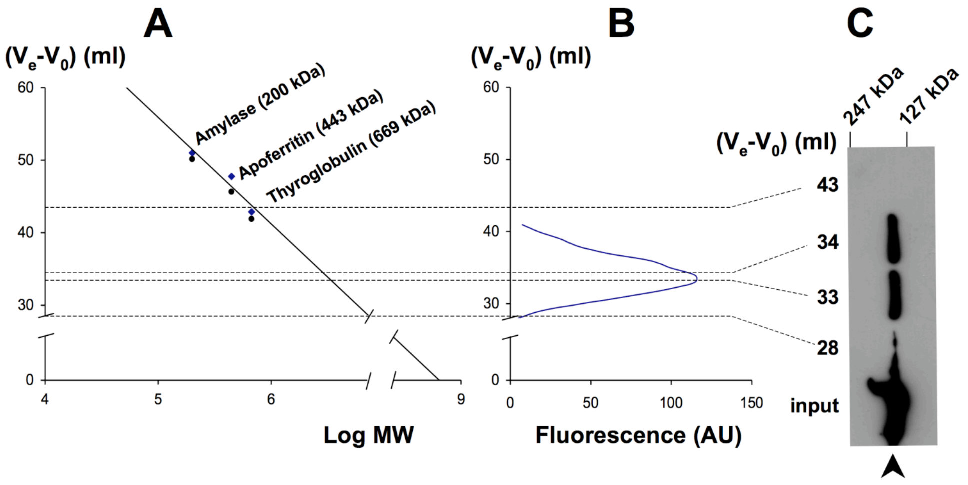

Figure S2. Gel permeation analysis of soluble extracts from *E. coli* producing PdhS-mCherry, combined with fluorescence and Western blot. For clarity, the void volume (V_0) was deduced from the elution volume (V_e). (A) The gel permeation column was calibrated using three proteins of known molecular weight (MW) as standards. When the soluble fraction of the bacterial cell lysate was loaded onto the column, a peak was observed, eluting at 33 and 34 ml (V_e-V_0). (B) These fractions, eluting at 33 and 34 ml, also correspond to a peak of fluorescence. The excitation wavelength (578 nm) and emission wavelength (612 nm) are characteristic of the mCherry fluorochrome. The fluorescence intensity is given in arbitrary units (AU). (C) A band at the expected molecular weight of the PdhS-mCherry fusion protein is also observed in Western blot for these fractions (arrowhead), while it less abundant or undetectable in fractions eluting at 28 and 43 ml, respectively. Anti-DsRed antibodies were used as in figure S1, and a sample of the soluble extract was load in the "input" lane. Altogether, these data suggest that PdhS-mCherry forms multimeric assemblies in solution with a predicted molecular weight comprised between 669 kDa and 20,000 kDa (upper limit of the fractionation range of the column used).
